# Regional Patterns in the Prevalence of Patient‐Reported Urticaria Aggravating Factors: A Multinational Mobile Health Study

**DOI:** 10.1002/clt2.70220

**Published:** 2026-11-08

**Authors:** Nuno Lourenço‐Silva, Ana Gimenez Arnau, Ivan Cherrez‐Ojeda, Jean Bousquet, Jonathan A. Bernstein, Luis Felipe Ensina, Silvia Mariel Ferrucci, Michihiro Hide, Andreas Kleinheinz, Emek Kocatürk, Philip H. Li, Markus Magerl, Aiste Ramanauskaite, Krzysztof Rutkowski, Petra Staubach, Rafael José Vieira, Ellen Witte‐Händel, Karsten Weller, Zuotao Zhao, Martin Metz, Sophia Neisinger, Bernardo Sousa Pinto

**Affiliations:** ^1^ MEDCIDS‐Department of Community Medicine, Information and Health Decision Sciences Faculty of Medicine University of Porto Porto Portugal; ^2^ Faculty of Medicine CINTESIS@RISE—Health Research Network University of Porto Porto Portugal; ^3^ Department of Dermatology, Urticaria Center of Reference, and Excellence (UCARE) Hospital del Mar Research Institute Universitat Pompeu Fabra Barcelona Spain; ^4^ Institute of Allergology Charité – Universitätsmedizin Berlin Corporate Member of Freie Universität Berlin and Humboldt‐Universität zu Berlin Berlin Germany; ^5^ Fraunhofer Institute for Translational Medicine and Pharmacology ITMP, Immunology and Allergology Berlin Germany; ^6^ Universidad Espíritu Santo Respiralab Research Center Guayaquil Ecuador; ^7^ Division of Rheumatology, Allergy and Immunology University of Cincinnati College of Medicine Cincinnati Ohio USA; ^8^ Department of Pediatrics Division of Allergy, Immunology and Rheumatology Federal University of São Paulo São Paulo Brazil; ^9^ Hospital Sírio‐Libanes‐São Paulo São Paulo Brazil; ^10^ Dermatology Section Fondazione IRCCS Ca’ Granda Ospedale Maggiore Policlinico Milan Italy; ^11^ Department of Dermatology Hiroshima City Hiroshima Citizens Hospital Hiroshima Japan; ^12^ Department of Dermatology Hiroshima University Hiroshima Japan; ^13^ Clinic for Dermatology Elbe Klinikum Buxtehude Buxtehude Germany; ^14^ Department of Dermatology Bahçeşehir University School of Medicine Istanbul Türkiye; ^15^ Department of Medicine Division of Rheumatology and Clinical Immunology University of Hong Kong Hong Kong China; ^16^ Urticaria Clinic St John’s Institute of Dermatology Guy’s and St Thomas’ Hospital London UK; ^17^ Departement of Dermatology Johannes Gutenberg, University University Medical Center Mainz Germany; ^18^ Department of Dermatology Tianjin Institute of Integrative Dermatology Tianjin Academy of Traditional Chinese Medicine Affiliated Hospital Tianjin China

**Keywords:** aggravating factors, chronic spontaneous urticaria, CRUSE, mobile health, multinational study

## Abstract

**Background:**

In chronic spontaneous urticaria (CSU), exacerbations are often associated with aggravating factors. However, the prevalence of patients having aggravating factors has been insufficiently studied. This mHealth study aimed to (i) estimate the prevalence of patient‐reported perceived aggravating factors for CSU across 10 countries, and (ii) identify factors associated with such triggers.

**Methods:**

We performed a cross‐sectional analysis of data from users of the CRUSE mobile application. The study included patients aged 15–74 years with self‐reported CSU. We assessed the frequency of reporting at least one of the following perceived aggravating factors: stress, food, exercise, low or high temperature, rubbing of the skin, infection, and medication. To correct for sample biases in age and sex distribution, we employed Multilevel Regression with Poststratification (MRP) models. In addition, we built logistic regression models to identify factors associated with perceived aggravating factor reporting.

**Results:**

Of the 12,243 included participants, 62.1% reported at least one perceived aggravating factor. Stress (31.1%) and rubbing of the skin (23.1%) were the most common. After MRP, the frequency of reporting at least one perceived aggravating factor ranged between 39.6% (Japan) and 83.7% (Brazil). The reporting frequency was lower in Asian than in Western countries. Female sex was independently associated with higher odds of reporting most perceived aggravating factors, except exercise and food. Younger age and lower disease control were associated with higher aggravating factor reporting.

**Conclusions:**

Patient‐reported perceived aggravating factors varied substantially across countries. Female sex, younger age, and poorer disease control were associated with increased reporting. Cross‐country differences may reflect biological, healthcare‐related, cultural, and psychosocial influences.

## Graphical abstract

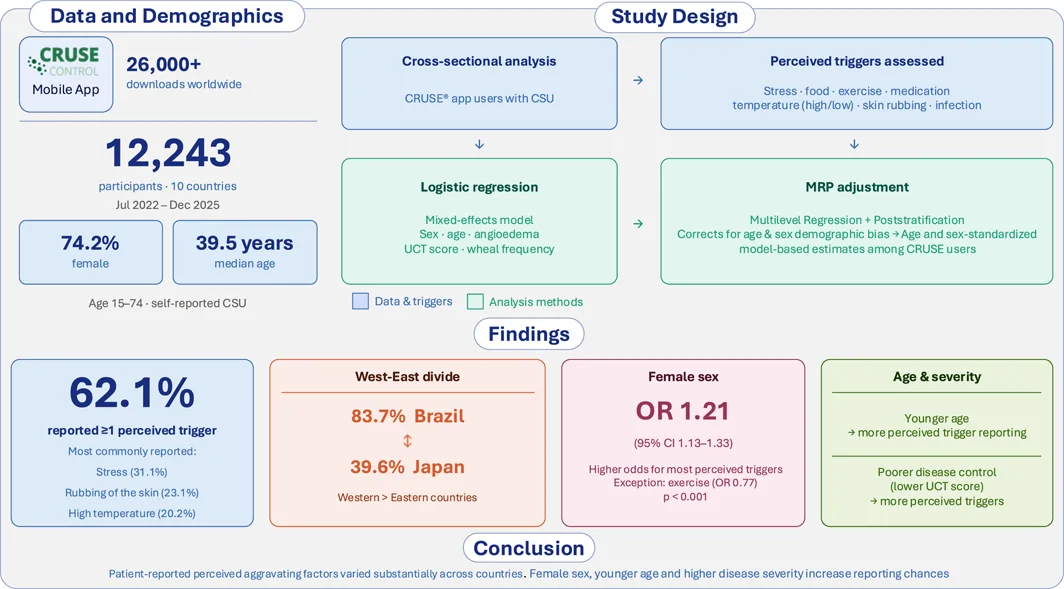

## 1 Introduction

Chronic spontaneous urticaria (CSU) is a debilitating inflammatory skin disorder characterised by the recurrent appearance of wheals, angioedema, or both, persisting for more than 6 weeks [1, 2]. The condition is associated with substantial impairment in quality of life, work productivity, and psychological wellbeing [1, 2, 3].

CSU is characterised by the spontaneous occurrence of wheals and/or angioedema in the absence of an identifiable external cause. Nevertheless, patients frequently attribute symptom worsening to a wide range of perceived aggravating factors [3, 4]. Some of the most commonly reported include physical, psychological, dietary, infectious, and medication‐related factors [1, 3, 5, 6, 7, 8]. For example, patients frequently report certain foods as CSU aggravating factors, even though there is currently no consensus to support food as a common causal aggravating factor of CSU. Patients also often report as CSU aggravating factors, specific and reproducible aggravating factors more characteristic of chronic inducible urticaria (CIndU), such as rubbing of the skin, exercise, or exposure to high or low temperatures. However, it should be noted that if these factors only occasionally (and not consistently) exacerbate symptoms, they may still represent unspecific aggravating factors within CSU.

The prevalence of CSU patients perceiving these factors as cause for worsening symptoms and their variation remains poorly characterised [4, 7, 9]. Mapping these perceptions is relevant to understanding how patients see their disease and to help healthcare practitioners provide adequate education. However, much of the existing evidence derives from single‐center studies or small cohorts, often limited by selection bias and lack of demographic representativeness. Mobile health (mHealth) platforms offer new opportunities to capture patient‐reported outcomes at scale, enabling the study of symptom patterns across diverse geographic regions. Nevertheless, app‐based datasets are inherently subject to demographic imbalances, as users are typically younger and more health‐engaged [10, 11, 12, 13]. Without appropriate adjustment, crude estimates derived from such data may not reflect population‐level patterns. Multilevel regression and poststratification (MRP) is a possible approach to address these limitations by combining individual‐level modelling with external demographic information to generate population‐adjusted estimates [14, 15]. This approach has been widely applied in epidemiological research to correct for imbalances in sample characteristics [15, 16, 17, 18, 19].

In this study, we utilised mHealth data from a multinational cohort of patients with CSU to obtain population‐adjusted estimates in patient‐reported perceived aggravating factors (i.e., patient‐reported perceived aggravating factors) while accounting for age‐ and sex‐related differences in the sampled populations. Additional objectives included (i) the comparison of regional patterns regarding the frequency of reported perceived aggravating factors, and (ii) the assessment of the association between the presence of reported perceived aggravating factors and demographic and clinical predictors.

## 2 Methods

### 2.1 Study Design

We conducted a cross‐sectional analysis of data obtained from the CRUSE mobile application. We started by evaluating the unadjusted frequency of CSU reported perceived aggravating factors among CRUSE users of each country. In addition, we evaluated whether there was an association between (i) the presence of reported perceived aggravating factors, and (ii) demographic and clinical characteristics. Finally, given that CRUSE users are not representative of the general patient population of patients with CSU, we employed MRP methods to obtain adjusted estimates on the frequency of reported perceived aggravating factors after correcting for sample biases. For MRP, country‐specific age‐ and sex‐distributions were obtained from published epidemiological studies reporting CSU prevalence in the general population. One representative study was selected for each country based on data availability and population coverage.

### 2.2 Setting and Participants

The CRUSE mobile application was launched in March 2022 and is currently available in 26 languages and 43 countries, with over 26,000 downloads [20, 21, 22]. The application is freely available on the Google Play and Apple App Stores, and has been used by patients who were referred by physicians and by patients who discovered the app themselves. It includes a daily monitoring questionnaire, in which patients can register their CSU symptoms and medication use. This allows patients to be more self‐aware of their disease and to share with healthcare professionals information about their level of disease control and medication adherence between consultations.

Patients are asked the following question during onboarding within the App: ‘Some patients with chronic spontaneous urticaria have aggravating factors, things that make their urticaria worse. Do you?’; those responding with yes can select one or more predefined factors (food, infection, medication, stress, exercise, cold temperatures, high temperatures, rubbing of the skin, or other), with the option for free‐text entry if ‘other’ is selected. This selection can be modified or expanded at any time by the patient in the profile.

While the app collects daily longitudinal monitoring data, our primary outcome corresponds to a profile‐level variable regarding perceived aggravating factors entered during onboarding or updated subsequently. Therefore, our analysis of these factors is inherently cross‐sectional (there is only one observation per patient regarding his/her aggravating factors). We included data from 12,243 participants registered between July 2022 and December 2025, aged 15–74 years, with a self‐reported diagnosis of CSU, and from Brazil, China, France, Germany, Italy, Japan, South Korea, Spain, United Kingdom, or USA. We restricted eligibility to these countries because, based on our literature search, they were the only ones with sufficient data available to support the application of MRP (i.e., the only countries for which it was possible to project the number of individuals of each age group and sex living with CSU). The age restrictions were related to the available data on the frequency of CSU per demographic group, something required for performing MRP. Participants missing essential demographic information required for poststratification, such as sex or age, were entirely excluded from the analysis.

### 2.3 Variables

The main outcome was the proportion of participants reporting one or more patient‐perceived aggravating factors (‘aggravating factors’ as labelled within the CRUSE application). In addition, we assessed the frequency of patients reporting each of the following perceived aggravating factors: exercise, low or high temperature, rubbing of the skin, food, infection, medication, and stress. All these perceived aggravating factors were self‐reported by the patients and no other exposure measurements were performed. Variables that were additionally evaluated included sex, age (stratified into 12 five‐year intervals: 15–19 to 70–74), presence of angioedema, frequency on the appearance of wheals, and the baseline Urticaria Control Test (UCT) score (a proxy of baseline CSU control).

### 2.4 Ethics

Ethical approval for the analysis of anonymised data was granted by the Ethics Committee of Charité‐Universitätsmedizin Berlin (EA4/184/23). Upon registration, all users consented to the anonymous use of their data for research purposes by accepting the app’s Terms and Conditions and Data Protection agreement. Data are stored in the Netherlands (CloudVPS) in compliance with the General Data Protection Regulation (EU) 2016/679 (GDPR), as well as ISO 27001 and ISO 13485 standards.

### 2.5 Statistical Analysis

Categorical variables were described using absolute and relative frequencies. Continuous variables were described using means and standard deviations or medians and interquartile ranges, as appropriate. All analyses were conducted in R (version 4.5.1), utilising the lme4 and arm packages for multilevel modelling and simulation.

### 2.6 Regression Models

Both for the presence of at least one reported perceived aggravating factor and for the presence of each individual perceived aggravating factor, we fitted logistic mixed‐effects regression models to identify associated factors. The presence of a reported perceived aggravating factor served as the dependent variable. Random effects were specified for the country, while sex, age, presence of angioedema, frequency of wheals episodes, and baseline UCT score were included as fixed effects. All models were checked for convergence and fit.

### 2.7 Multilevel Regression Model With Poststratification

To obtain adjusted estimates of the prevalence of each reported perceived aggravating factor correcting for sampling biases, we employed MRP methods (see Supporting Information S1: Methods for a more detailed description). In brief, this implied projecting the number of individuals living with CSU per age group and sex in each country. Therefore, we constructed a post‐stratification frame based on census data for the adult population (aged 15–74) of each of the 10 included countries. Details of the selected epidemiological sources and respective methodology for each included country are provided in Supporting Information S1: Table S1. To account for uncertainty in the model parameters, 1000 simulations of the model coefficients were performed.

## 3 Results

### 3.1 Study Population and Descriptive Characteristics

Of 27,165 CRUSE users, a total of 12,243 participants with self‐reported CSU were included in the final analysis (Supporting Information S1: Figure S1). The study population was predominantly female (74.2%, *n* = 9081), with a median age of 38 (IQR 29–48) years. Baseline demographic and clinical characteristics are presented in Table 1.

**TABLE 1:** Characteristics of the assessed participants.

Variable	Assessed sample (*n* = 12,243)
Age (median, IQR)	38 (29–48)
Female (*n*, %)	9081 (74.2)
Reporting of perceived aggravating factors (*n*, %)	7599 (62.1)
Exercise	1324 (10.8)
Low temperature	1020 (8.3)
High temperature	2479 (20.2)
Rubbing of the skin	2830 (23.1)
Food‐related	2030 (16.6)
Medication	1254 (10.2)
Infection	1099 (9.0)
Stress	3808 (31.1)
Angioedema (*n*, %)	6005 (49.0)
Frequency on the appearance of wheals (*n*, %)	
Every day	4619 (37.7)
Almost every day	3110 (25.4)
Once or more than once per week	2598 (21.2)
Once per month or less than once per month	1567 (12.8)
Never	349 (2.9)
Baseline UCT (median, IQR)	6 (4–8)

Abbreviations: IQR = Interquartile Range; UCT = Urticaria Control Test.

Most participants (*n* = 7599; 62.1%) reported at least one perceived aggravating factor. Stress was the most frequently reported perceived aggravating factor (31.1%), followed by rubbing of the skin (23.1%), high temperature (20.2%), and food (16.6%). Exercise (10.8%), medication (10.2%), infections (9.0%), and low temperature (8.3%) were reported less frequently.

Country‐level data (Supporting Information S1: Table S2) showed that participants’ mean age ranged from 37.5 years in Brazil to 42.1 years in Italy, and that women predominated in all countries (ranging from 66.1% in Japan to 79.3% in Brazil). The unadjusted percentage of participants reporting at least one perceived aggravating factor varied substantially, ranging from 39.7% in Japan and 43.2% in South Korea to 84.6% in Brazil.

### 3.2 Associations With Age, Sex, and Clinical Characteristics

Table 2 summarises the results of the multivariable mixed‐effects logistic regression models evaluating associations between demographic and clinical variables and reporting of at least one reported perceived trigger or specific perceived aggravating factors.

**TABLE 2:** Results of the multivariable logistic regression models evaluating the association between sex and age, and (i) reporting at least one perceived aggravating factor (‘any aggravating factor’) or individual urticaria perceived aggravating factors.

Association‐odds ratio (95% CI), *p*‐value	Perceived aggravating factor								
	Any aggravating factor	Stress	Low temperature	High temperature	Rubbing of the skin	Food	Infection	Medication	Exercise
Female sex	1.21 (1.13–1.33) *p* < 0.001	1.24 (1.13–1.37) *p* < 0.001	1.40 (1.18–1.65) *p* < 0.001	1.13 (1.01–1.26) *p* = 0.036	1.57 (1.41–1.75) *p* < 0.001	1.05 (0.93–1.18) *p* = 0.470	1.53 (1.29–1.82) *p* < 0.001	1.59 (1.35–1.88) *p* < 0.001	0.77 (0.67–0.87) *p* < 0.001
Age	0.99 (0.98–0.99) *p* < 0.001	0.99 (0.98–0.99) *p* < 0.001	0.98 (0.97–0.98) *p* < 0.001	0.98 (0.98–0.99) *p* < 0.001	0.99 (0.98–0.99) *p* < 0.001	0.99 (0.98–0.99) *p* = 0.015	0.99 (0.98–0.99) *p* = 0.002	1.01 (1.00–1.01) *p* = 0.057	0.97 (0.96–0.97) *p* < 0.001
Angioedema	1.14 (1.05–1.24) *p* = 0.001	1.40 (1.29–1.52) *p* < 0.001	1.14 (0.99–1.31) *p* = 0.069	1.00 (0.91–1.10) *p* = 0.995	1.12 (1.02–1.22) *p* = 0.017	1.42 (1.28–1.57) *p* < 0.001	2.44 (2.12–2.81) *p* < 0.001	2.39 (2.09–2.73) *p* < 0.001	1.00 (0.88–1.13) *p* = 0.945
Baseline UCT score	0.98 (0.96–0.99) *p* < 0.001	0.98 (0.97–0.99) *p* < 0.001	0.99 (0.97–1.01) *p* = 0.18	0.96 (0.95–0.97) *p* < 0.001	0.99 (0.98–1.00) *p* = 0.073	0.97 (0.95–0.98) *p* < 0.001	0.98 (0.96–0.99) *p* = 0.019	0.97 (0.96–0.99) *p* = 0.002	0.97 (0.95–0.98) *p* < 0.001
Frequency of wheals									
Almost every day	1.23 (1.11–1.36) *p* < 0.001	1.14 (1.02–1.26) *p* = 0.017	1.31 (1.10–1.56) *p* = 0.002	1.10 (0.98–1.24) *p* = 0.105	0.92 (0.83–1.03) *p* = 0.167	1.05 (0.92–1.19) *p* = 0.476	1.01 (0.85–1.20) *p* = 0.938	1.13 (0.96–1.32) *p* = 0.147	1.22 (1.05–1.42) *p* = 0.009
Once a week	1.40 (1.25–1.56) *p* < 0.001	1.13 (1.00–1.26) *p* = 0.043	1.53 (1.27–1.84) *p* < 0.001	1.10 (0.96–1.25) *p* = 0.166	0.92 (0.82–1.04) *p* = 0.204	1.06 (0.92–1.22) *p* = 0.460	1.08 (0.90–1.30) *p* = 0.433	1.10 (0.92–1.32) *p* = 0.279	1.21 (1.02–1.44) *p* = 0.028
Once a month	1.51 (1.31–1.74) *p* < 0.001	1.19 (1.03–1.37) *p* = 0.019	1.43 (1.12–1.81) *p* = 0.003	1.08 (0.92–1.28) *p* = 0.352	0.72 (0.61–0.84) *p* < 0.001	1.16 (0.97–1.39) *p* = 0.103	1.57 (1.26–1.95) *p* < 0.001	1.51 (1.23–1.87) *p* < 0.001	1.24 (1.00–1.54) *p* = 0.056
Never	0.67 (0.48–0.94) *p* = 0.020	0.53 (0.34–0.82) *p* = 0.004	0.61 (0.27–1.40) *p* = 0.245	0.54 (0.31–0.93) *p* = 0.027	0.46 (0.28–0.75) *p* = 0.002	0.57 (0.32–1.02) *p* = 0.058	0.48 (0.20–1.20) *p* = 0.116	0.58 (0.27–1.27) *p* = 0.174	0.75 (0.39–1.46) *p* = 0.400

Abbreviations: CI = Confidence Internal; UCT = Urticaria Control Test.

Female sex was associated with increased odds of reporting at least one reported perceived aggravating factor (OR = 1.21, 95% CI = 1.13–1.33, *p* < 0.001). Women were also more likely to perceive stress (OR = 1.24, 95% CI = 1.13–1.37), low temperature (OR = 1.40, 95% CI = 1.18–1.65), high temperature (OR = 1.13, 95% CI = 1.01–1.26), rubbing of the skin (OR = 1.57, 95% CI = 1.41–1.75), infection (OR = 1.53, 95% CI = 1.29–1.82), and medication as aggravating factors (OR = 1.59, 95% CI = 1.35–1.88; all *p* < 0.001). No significant association was observed between female sex and food‐related perceived aggravating factors. In contrast, perception of exercise‐related aggravating factors were less frequently reported by females (OR = 0.77, 95% CI = 0.67–0.87, *p* < 0.001).

Increasing age was consistently associated with lower odds of reporting any reported perceived aggravating factor (OR = 0.99 per year, 95% CI = 0.98–0.99, *p* < 0.001) and most individual perceived aggravating factors, including stress, temperature, rubbing of the skin, infection, and exercise. Age was not significantly associated with perception of medication aggravating factors.

The presence of angioedema was associated with higher odds of reporting any reported perceived aggravating factor (OR = 1.14, 95% CI = 1.05–1.24), stress (OR = 1.40, 95% CI = 1.29–1.52), rubbing of the skin (OR = 1.12, 95% CI = 1.02–1.22), food (OR = 1.42, 95% CI = 1.28–1.57), infection (OR = 2.44, 95% CI = 2.12–2.81), and medication (OR = 2.39, 95% CI = 2.09–2.73).

Higher UCT scores, reflecting better disease control, were consistently associated with lower odds of reporting perceived aggravating factors across most domains. Similarly, greater wheal frequency was associated with increased odds of reporting any perceived aggravating factor and several individual perceived aggravating factors, particularly low temperature and exercise, while patients reporting no wheals demonstrated lower odds of perceived aggravating factor reporting.

### 3.3 Prevalence of Reported Aggravating Factors

Table 3 presents the results of the adjusted estimates of the frequency of reported perceived aggravating factors after using MRP to correct for sample bias. The frequency of patients reporting at least one reported perceived aggravating factor ranged from 39.6% (Japan) to 83.7% (Brazil). Overall, the frequency of patients reporting perceived aggravating factors was lower in Asian countries (ranging from 39.6% in Japan to 52.7% in China) than in European (from 57.7% in Spain to 62.2% in France) or American countries (61.0% in the USA and 83.7% in Brazil). This discrepancy was particularly observed for stress and physical aggravating factors. Stress remained the most prevalent perceived aggravating factor in all regions, with adjusted estimates ranging from 20.9% (95% CI = 17.2–25.2%) in Japan to 38.6% (95% CI = 33.4–44.1%) in France.

**TABLE 3:** Age‐ and sex‐adjusted estimates of the frequency of reported perceived aggravating factors among CRUSE users.

Reporting of the perceived aggravating factor‐median % (95% CI)	Brazil	China	France	Germany	Italy	Japan	Korea	Spain	UK	USA
At least one aggravating factor	83.7 (81.8–85.3)	52.7 (43.8–61.3)	62.2 (55.9–67.7)	58.4 (56.4–60.5)	60.4 (55.8–64.9)	39.6 (34.7–45.5)	42.3 (37.9–46.5)	57.7 (54.2–61.2)	60.9 (58.2–63.4)	61.0 (58.7–63.2)
Stress	35.3 (33.1–37.5)	21.8 (16.4–28.8)	38.6 (33.4–44.1)	27.5 (25.6–29.6)	28.0 (24.5–32.0)	20.9 (17.2–25.2)	22.2 (18.8–25.8)	27.9 (24.9–31.4)	32.7 (30.4–35.1)	32.2 (30.0–34.4)
Low temperature	5.9 (4.9–7.2)	6.3 (3.9–9.4)	10.4 (7.6–14.3)	8.4 (7.2–10.1)	6.4 (4.6–8.9)	4.8 (3.2–7.1)	8.2 (6.1–10.6)	7.3 (5.6–9.3)	12.5 (10.9–14.5)	8.4 (7.0–9.8)
High temperature	21.1 (19.3–23.1)	11.9 (7.9–16.8)	21.1 (16.6–26.1)	18.6 (16.8–20.6)	16.8 (13.8–20.3)	10.6 (7.8–14.0)	11.5 (9.2–14.4)	17.3 (14.6–20.4)	28.2 (25.8–30.6)	24.7 (22.5–27.3)
Rubbing of the skin	22.5 (20.4–24.4)	16.7 (12.4–21.4)	22.5 (18.0–27.2)	21.4 (19.4–23.4)	20.0 (16.8–23.4)	14.2 (11.3–17.8)	14.4 (11.8–17.7)	15.3 (13.0–17.9)	22.4 (20.1–24.5)	25.0 (22.8–27.3)
Food‐related	15.7 (14.1–17.4)	16.5 (12.0–21.5)	16.9 (13.2–21.3)	15.7 (14.3–17.0)	19.1 (15.8–22.8)	9.8 (7.3–13.0)	15.1 (12.4–18.5)	9.9 (8.1–12.0)	18.5 (16.7–20.5)	17.2 (15.4–19.0)
Infection	7.2 (6.0–8.5)	4.7 (2.3–9.0)	11.0 (7.8–15.2)	9.8 (8.7–11.2)	6.0 (4.1–8.4)	1.8 (0.8–3.3)	2.9 (1.8–4.5)	3.3 (2.4–4.8)	10.3 (8.9–11.9)	6.4 (5.4–7.7)
Medication	16.1 (14.2–17.9)	9.0 (5.2–14.1)	11.8 (8.6–15.6)	9.1 (8.1–10.2)	12.5 (9.8–15.7)	3.6 (2.1–5.9)	5.0 (3.4–7.1)	5.6 (4.2–7.3)	7.2 (6.0–8.6)	6.9 (5.9–8.0)
Exercise	10.5 (9.1–11.9)	7.9 (5.3–11.4)	13.8 (10.4–17.8)	11.7 (10.0–13.9)	10.9 (8.3–13.9)	6.2 (4.3–8.7)	8.0 (6.1–10.3)	9.7 (7.6–12.2)	17.8 (15.5–19.9)	14.1 (12.2–16.3)

Abbreviations: CI = Confidence interval; UK = United Kingdom; USA = United States of America.

The frequency of patients perceiving high temperature exposure as an aggravating factor ranged from 10.6% (95% CI = 7.8–14.0%) in Japan to 28.2% (95% CI = 25.8–30.6%) in the United Kingdom, while low temperature were less frequently perceived overall, ranging from 4.8% (95% CI = 3.2–7.1%) in Japan to 12.5% (95% CI = 10.9–14.5%) in the United Kingdom. Rubbing of the skin varied from 14.2% (95% CI = 11.3–17.8%) in Japan to 25.0% in the United States.

Exercise‐related reported perceived aggravating factors were less frequently reported than stress or physical factors, having their lowest prevalence in Japan (6.2%, 95% CI = 4.3–8.7%) and China (7.9%, 95% CI = 5.3–11.4%) and the highest in the United States (14.1%, 95% CI = 12.2–16.3%) and in the United Kingdom (17.8%, 95% CI = 15.5–19.9%). On the other hand, the percentage of patients reporting food perceived aggravating factors ranged from 9.8% (95% CI = 7.3–13.0%) in Japan and 9.9% (95% CI = 8.1–12.0%) in Spain to 18.5% (95% CI = 16.7–20.5%) in the United Kingdom and 19.1% (95% CI = 15.8–22.8%) in Italy.

Infection and medication‐related reported perceived aggravating factors were consistently less prevalent across countries. Infection ranged from 1.8% (95% CI = 0.8–3.3%) in Japan to 11.0% (95% CI = 7.8–15.2%) in France, while medication varied from 3.6% (95% CI = 2.1–5.9%) in Japan to 16.1% (95% CI = 14.2–17.9%) in Brazil.

Among users reporting at least one reported perceived aggravating factor (Supporting Information S1: Table S3), stress remained the most frequent one across all countries, ranging from 42.1% (95% CI = 38.1–46.6%) in China to 60.3% (95% CI = 55.8–64.7%) in France. High temperature was the most common physical perceived aggravating factor, varying from 23.1% (95% CI = 20.6–25.9%) in China to 45.6% (95% CI = 41.1–49.7%) in the United Kingdom, whereas low temperature was less frequently reported (7.1% in Brazil to 20.8% in the United Kingdom). Rubbing of the skin was consistently reported in approximately one‐third of users, reaching 40.6% (95% CI = 36.1–45.2%) in the United States. Exercise and food‐related perceived aggravating factors showed moderate variability across countries, while infection and medication were generally less common, particularly in Japan (4.5% and 9.9%, respectively).

## 4 Discussion

In this mHealth international study, we observed substantial across‐country variability in the frequency of reported perceived CSU aggravating factors. To our knowledge, this represents the largest international assessment on such perceptions. We observed marked differences in reporting patterns between users from Western and Eastern countries, with reported perceived aggravating factors being more commonly reported in Western countries. Such differences, are unlikely to reflect biological variation alone. Because the assessed factors were patient‐perceived rather than clinically validated aggravating factors, cross‐country differences may also reflect variation in symptom attribution, illness perception, and cultural beliefs regarding disease exacerbation [23, 24]. Similar to findings in other inflammatory skin conditions, where these factors have been shown to impact patient‐reported outcome measures across distinct geographic populations, these elements also likely shape how patients with CSU recognise and report their aggravating factors [23, 24, 25, 26].

Considering individual reported perceived aggravating factors, stress emerged as the most prevalent one across all countries, followed by physical factors such as rubbing of the skin and high temperature. Female sex, younger age and a less well controlled condition (lower UCT baseline score) were independently associated with a higher likelihood of reporting most perceived aggravating factors. Stress was consistently identified as the most frequently reported perceived aggravating factor, reinforcing its well‐established role in CSU exacerbations [5, 24, 27, 28, 29]. Psychological stress is known to modulate mast cell activity through neuroimmune pathways, contributing to disease flares [5, 6, 28]. This occurs as stress‐induced neurotransmitters aggravating factor the degranulation of nearby dermal mast cells and promote the recruitment of inflammatory cells like neutrophils and eosinophils [2, 6, 30]. Physical perceived aggravating factors were also commonly reported but their frequency demonstrated marked geographic variability [1, 2, 24, 28, 31, 32]. This variability likely reflects a combination of climatic differences, occupational exposure, lifestyle factors, and cultural interpretation of symptoms [2, 32]. In addition, in some cases, these factors may reflect concomitant CIndU, including symptomatic dermographism, cold urticaria, heat urticaria, or cholinergic urticaria, which frequently coexist with CSU. Because CRUSE does not distinguish spontaneous from CIndU or does not allow to identify patients with coexisting CindU (even though it was designed for patients with CSU), these findings should be interpreted cautiously.

The prevalence of food‐related perceived aggravating factors also varied considerably between countries. While true IgE‐mediated food allergy is uncommon in CSU, perceived food‐related exacerbations are frequently described and may reflect pseudoallergic reactions, intolerance to food additives, or individual dietary sensitivities [5, 33, 34, 35]. The less commonly reported perceived aggravating factors were infection and medications, which also showed relevant geographic heterogeneity. This variation may be influenced by differences in healthcare access, prescription patterns, medication exposure, and infectious disease burden [5, 6, 7, 36]. Additionally, medication‐related reports likely represent a heterogeneous group, including medications known to exacerbate CSU (e.g., NSAIDs) as well as medications that patients perceive to coincide with the natural fluctuation of disease activity [37].

Regarding key demographics, the observed sample of CRUSE users is consistent with that of previous established CSU cohorts, underscoring that CRUSE users may not be so demographically different from overall CSU patients. Analyses revealed consistent sex‐related differences in aggravating factor reporting. Female sex was associated with increased odds of reporting most perceived aggravating factors, particularly rubbing of the skin, infection, and medication. This pattern aligns with prior observations of greater CSU prevalence, disease burden, and symptom reporting among women [26, 31, 38, 39]. Potential explanations include hormonal influences on mast cell reactivity, sex‐specific immune responses, and behavioural factors such as heightened symptom awareness or healthcare‐seeking behaviour [26, 38]. In contrast, the only perceived aggravating factor that was more frequently reported by males was that of exercise, which may reflect differences in physical activity patterns or exposure to exertional stimuli rather than intrinsic biological susceptibility [38, 40].

Increasing age was consistently associated with lower reporting of most reported perceived aggravating factors. This trend may reflect reduced environmental exposure, altered immune responsiveness with ageing, and differences in symptom perception or disease understanding [5, 7, 28, 41].

Overall, perceived aggravating factor reporting was associated not only with demographic characteristics but also with markers of disease activity and control. The observed association between angioedema and increased likelihood of reporting aggravating factors may reflect, at least in part, a perception‐driven phenomenon rather than a purely biological relationship. Patients experiencing angioedema, which is often more distressing and clinically impactful than wheals alone, may be more likely to actively search for explanations or external factors associated with symptom worsening [25, 42, 43]. This heightened attribution tendency could lead to increased reporting of potential aggravating factors.

Similarly, poorer disease control, as indicated by lower UCT scores, was consistently associated with higher odds of perceived aggravating factor reporting across most domains. This suggests that patients experiencing uncontrolled symptoms may be more vigilant in attributing flares to external or internal stimuli, potentially amplifying perceived aggravating factor effects.

Taken together, these findings suggest that geographic differences in reported aggravating factors likely reflect a combination of biological, environmental, healthcare‐related, and psychosocial influences. Rather than simply indicating true differences in the prevalence of causal aggravating factors or mirroring variations in exposure levels to different factors, the observed variability may also capture differences in how patients from different cultural settings recognize, interpret, and attribute symptom worsening. This interpretation is consistent with the substantial between‐country variation observed for factors such as food, for which current evidence supporting a causal role in CSU is limited.

A key strength of this study lies in its sample size and wide geographic distribution, allowing for the comparison of regional patterns of aggravating factor reporting. Additionally, the use of MRP allowed us to adjust for demographic imbalances inherent to app‐based samples and to generate nationally representative prevalence estimates.

Several limitations should be acknowledged. First, all data were self‐reported, introducing potential misclassification bias. Not all CSU diagnoses were physician‐verified. Consequently, we were unable to distinguish CSU with coincidental aggravating factor exposure from true comorbid CIndU phenotypes, particularly for physical stimuli such as heat, cold, and rubbing of the skin. Because of this absence of information, we could not perform a sensitivity analysis excluding CIndU phenotypes. Although MRP reduced imbalance with respect to age and sex, it cannot account for residual selection bias related to app use, healthcare access, disease control, socioeconomic characteristics, or health‐seeking behaviour. Second, specific inducible subtypes such as cholinergic urticaria could not be reliably differentiated, and exercise‐related symptoms may reflect heterogeneous mechanisms, including thermoregulatory responses rather than direct trigger effects. Third, while CRUSE registers the factors that patients perceive as aggravating factors, it does not assess, in its daily monitoring questionnaire, whether patients have been exposed to them on each day. Therefore, the app is not able to capture whether exposure to reported perceived aggravating factors is associated with new lesion onset, worsening of existing wheals, angioedema episodes, or merely increased pruritus. This is particularly important as patient interpretations of what constitutes an ‘aggravating factor’ may vary substantially, reflecting differences in symptom perception rather than distinct pathophysiological mechanisms. Additionally, regarding exercise‐related reports, the frequency of reporting may reflect differences in exposure to physical activity, sweating, occupational exertion, or environmental heat, rather than true biological differences in susceptibility to symptom aggravation. Similarly, we were not able to obtain information of what type(s) of infection or specific foods patients perceive as resulting in CSU worsening. Another aspect that is not reported in the dataset is that of patient ethnicity, which may in part explain differences in aggravating factor reporting. Fourth, sample sizes varied substantially across countries, being smaller in China and France, which may limit the precision of estimates. Although MRP adjusts for age and sex distributions, important unmeasured confounders remain unaddressed. These include disease control, health‐seeking behaviour, healthcare access, comorbidities, and treatment status. Furthermore, participation in a mobile health application likely selects for participants who are more engaged in symptom monitoring, which may amplify aggravating factor reporting. Finally, the cross‐sectional design precludes causal inference, and reported associations should be interpreted as descriptive patterns rather than mechanistic relationships.

In conclusion, this multinational mHealth‐based study demonstrates substantial geographic variability in the frequency of patient‐reported perceived aggravating factors in CSU, with such being higher in Western than in Eastern countries. In addition, this study identified female, younger age and poorer disease control as consistently associated with higher perceived aggravating factor reporting. These findings highlight the importance of discussing patient‐perceived aggravating factors during clinical assessment. Exploring patients’ beliefs regarding symptom worsening may help clinicians contextualise perceived aggravating factors, provide appropriate education, and address misconceptions while maintaining evidence‐based management of CSU.

## Author Contributions

**Nuno Lourenço‐Silva:** conceptualization, data curation, formal analysis, visualization, writing – original draft, methodology, investigation, project administration, validation, writing – review and editing. **Ana Gimenez Arnau:** writing – review and editing. **Ivan Cherrez‐Ojeda:** writing – review and editing. **Jean Bousquet:** writing – review and editing. **Jonathan A. Bernstein:** writing – review and editing. **Luis Felipe Ensina:** writing – review and editing. **Silvia Mariel Ferrucci:** writing – review and editing. **Michihiro Hide:** writing – review and editing. **Andreas Kleinheinz:** writing – review and editing. **Emek Kocatürk:** writing – review and editing. **Philip H. Li:** writing – review and editing. **Markus Magerl:** writing – review and editing. **Aiste Ramanauskaite:** writing – review and editing. **Krzysztof Rutkowski:** writing – review and editing. **Petra Staubach:** writing – review and editing. **Rafael José Vieira:** conceptualization, data curation, formal analysis, visualization, methodology, investigation, writing – review and editing, supervision, and validation. **Ellen Witte‐Händel:** writing – review and editing. **Karsten Weller:** writing – review and editing. **Zuotao Zhao:** writing – review and editing. **Martin Metz:** conceptualization, methodology, investigation, supervision, and writing – review and editing. **Sophia Neisinger:** conceptualization, data curation, formal analysis, visualization, writing – original draft, methodology, investigation, supervision, project administration, writing – review and editing, and validation. **Bernardo Sousa Pinto:** conceptualization, data curation, formal analysis, visualization, writing – original draft, methodology, investigation, supervision, project administration, writing – review and editing, and validation.

## Funding

The authors have nothing to report. The CRUSE App is supported by Novartis, Celldex and Moxie.

## Conflicts of Interest

Nuno Lourenço‐Silva has no conflict of interest to declare in relation to this work. Ana Gimenez‐Arnau has received consulting fees from Almirall, Amgen, Blue‐Print, CELLDEX, ESCIENT, FAES, Genentech, GSK, Jaspers, Leo Pharma, Mitsubishi Tanabe, Novartis, Noucor, Sanofi–Regeneron, Thermo Fisher Scientific, Septerna, Servier, Uriach Pharma; grants or contracts with ESCIENT, NOUCOR, Novartis, Instituto Carlos III–FEDER, Uriach Pharma; and payments or honoraria for lectures from Almirall, Avene, Genentech, GSK, LEO‐PHARMA, Menarini, MSD, NOUCOR, Novartis, Sanofi, Uriach Pharma. Ivan Cherrez‐Ojeda has no conflict of interest to declare in relation to this work. Jean Bousquet has no conflict of interest to declare in relation to this work. Jonathan A. Bernstein reports personal fees and grants from Sanofi Regeneron, AZ, Allakos, Celldex, Escient, Novartis, Genentech, Jasper Pharma, and roles as AAAAI immediate past president, WAO board member, AAAAI Foundation Chairperson, UCARE/ACARE centre member, and JTF practice parameters committee member. Luis Felipe Ensina declares consulting fees not related with this work from Sanofi and Lilly; honoraria for lectures from Sanofi, Novartis, Celldex and Celltrion; and travel grants from Sanofi. Silvia Mariel Ferrucci SMF, with no relation to this study, has been a speaker, advisory board members or Principal Investigator in clinical trials for: AbbVie, Almirall, Amgen, Incyte, Novartis, Pfizer, Leo Pharma, Sanofi, Elli Lilly, and Galderma. Michihiro Hide declares consulting fees from Japan Tobacco, Novartis, Sanofi, Taiho, Teikoku Seiyaku, and Yuhan; payments or honoraria for lectures from Kaken Pharmaceutical, Kyorin Pharmaceutical, Mitsubishi Tanabe Pharma, Meiji Seiyaku, Novartis, Sanofi/Regeneron, and TAIHO Pharmaceutical; and travel grants from TAIHO Pharmaceutical. Andreas Kleinheinz reports study funding, medical writing, honoraria, expert testimony, support for attending meetings/ travel, and participation on a data safety monitoring board or advisory board from Aimmune Therapeutics, a Nestlé Health Science company. Other disclosures include AbbVie, Aimmune Therapeutics, a Nestlé Health Science company, Almirall, Galderma, Janssen/JNJ, Leo Pharma, Lilly, Medac, Novartis, and Regeneron/Sanofi. Emek Kocatürk reports personal fees from Novartis, Menarini, outside the submitted work. Philip Li has no conflict of interest to declare in relation to this work. Markus Magerl is an advisor for MOXIE. Aiste Ramanauskaite has no conflict of interest to declare in relation to this work. Krzysztof Rutkowski has no conflict of interest to declare in relation to this work. Petra Staubach has acted as advisor/consultant for AbbVie, Advanz, Allergika, Almirall‐Hermal, Amgen, Beiersdorf, Biocryst, Biogen Idec, BMS, Boehringer‐Ingelheim, Celgene, CSL‐Behring, Eli‐Lilly, Galderma, Hexal, Ionis, Janssen, Kalvista, Klinge, LEO‐Pharma, LETI‐Pharma, L’Oréal, Novartis, Octapharma, Pfizer, Pflüger, Pharming, Regeneron, Shire, Takeda, Regeneron, Sanofi‐Genzyme UCB Pharma, Widmer. Rafael José Vieira has no conflict of interest to declare in relation to this work. Ellen Witte‐Händel has no conflict of interest to declare in relation to this work. Karsten Weller declares grants or contracts from Novartis, Sanofi, and Noucor, and consulting fees and advisory board participation for Novartis. Zuotao Zhao is a speaker/advisor for and/or has received research funding from Novartis, Sanofi, Pfizer, Astellas, Galderma, Janssen, GSK, Bayer, LEO, MEDA Pharma, and ALK Pharma. Martin Metz has received honoraria as a speaker and/or advisor from AbbVie, Advanz, ALK Abelló, Allegria, Almirall, Amgen, Argenx, Astra Zeneca, Astria, Attovia, Bambusa, Berlin‐Chemie, Blueprint, Celldex, Celltrion, DeepApple, Enanta, Escient, FatiAbGen, Ga2len, Galderma, Granular, GSK, Incyte, Japan Tobacco, Jasper, Lilly, Lycia, Novartis, Pfizer, Pharvaris, Proinnovera, Regeneron, Sanofi, Santa Ana Bio, Septerna, Teva, Third Harmonic Bio, Unna Akademie, Vifor. Sophia Neisinger has been a speaker/advisor for Novartis, Sanofi, Celltrion, Biocryst and Bencard. Bernardo Sousa Pinto has no conflict of interest to declare in relation to this work.

## Supporting information


Supporting Information S1

## Data Availability

The data that support the findings of this study are available on request from the corresponding author. The data are not publicly available due to privacy or ethical restrictions.
