# Supplementary material for: Regional Patterns in the Prevalence of Patient‐Reported Urticaria Aggravating Factors: A Multinational Mobile Health Study
Source: Clin Transl Allergy. 2026 Nov 8;16(11):e70220. doi: 10.1002/clt2.70220 (PMC13745295; doi:10.1002/clt2.70220)
Supplement: Supplementary file 1 [file CLT2-16-e70220-s001.docx]

**Supplementary Material**

**Supplementary Methods**

To correct for demographic imbalance inherent to mobile application data and generate age- and sex-standardized model-based estimates of chronic spontaneous urticaria (CSU) perceived aggravating factors, we applied a Multilevel Regression with Poststratification (MRP) framework. This approach combines hierarchical regression modelling with external population distributions to project individual-level app data onto known age-sex structures of the general population. Separate MRP models were fitted for each perceived aggravating factor.

**Construction of the Supporting Frame for MRP**

The reference data sources used to estimate the number of patients with CSU of each sex and age group in each country are detailed in Supplementary Table 1. While information on the national prevalence of CSU and on sex distribution of CSU patients was directly obtained from the source references for all countries, the methodology for obtaining information on the age distribution of patients with CSU varied depending on the granularity of the available data:

(i) Proportion-Based Derivation (Direct Extraction) - For countries where the reference literature provided explicit frequency distributions per specific age category (Brazil, China, and South Korea), we directly utilized these data.

(ii) Parametric Simulation - For countries where the reference literature provided only the mean and standard deviation age for patients with CSU without a detailed breakdown by age group (France, Germany, Italy, Japan, Spain, United Kingdom, and the United States of America), we estimated the number of participants with CSU of each age group by fitting probability distributions. In particular, we modelled the distribution of participants’ age using a Gamma distribution. We defined the shape and rate parameters of this distribution based on the reported mean and standard deviation from the literature using the method of moments. Finally, we categorized the obtained simulations into the study’s age intervals to calculate the estimated number of patients with CSU of each age group in each country.

Age was stratified into twelve 5-year intervals (from 15-19 to 70-74 years), and sex was coded as a binary variable. Therefore, we were able to obtain 24 demographic strata per country (male/female × 12 age groups). For each of these strata (i.e., each combination of a sex and age group), we not only had the projections on the number of CSU patients, but also on the total number of individuals in each country. Such information was obtained from government or United Nations databases (Supplementary Table 1)

**Assessment of uncertainty**

To propagate uncertainty from the fitted multilevel models into the final prevalence estimates, we generated 1,000 simulated draws from the joint sampling distribution of the model parameters using the arm package. For each simulation, linear predictors were constructed for all 24 demographic strata within each country by combining the global intercept with the simulated random effects for sex, age group, and country.

Country-level age- and sex-adjusted estimates were obtained by calculating weighted averages of these predicted probabilities across all demographic strata, using the corresponding age- and sex-specific population proportions for each country.

This procedure resulted in 1,000 simulated estimates for each aggravating factor and country. Final point estimates were calculated as the mean of these simulated distributions, while 95% uncertainty intervals were derived from the 2.5th and 97.5th percentiles.

**Supplementary Figures**

**Supplementary Figure 1. Flow diagram illustrating participants selection**

**Supplementary Tables**

| **Country** | **Reference source** | **Demographic data source** | **Methodology** |
| --- | --- | --- | --- |
| **Brazil** | Balp et al.^1^ | United Nations data^2^ | Proportion-based estimates were derived by normalizing category frequencies to cohort-specific sample sizes. |
| **China** | Zhang et al.^3^ | United Nations data^2^ | Proportion-based estimates were derived by normalizing category frequencies to cohort-specific sample sizes. |
| **France** | Balp et al.^4^ | Government data^5^ | Age categories were derived from Gamma-simulated distributions based on reported summary statistics |
| **Germany** | Balp et al.^4^ | United Nations data^2^ | Age categories were derived from Gamma-simulated distributions based on reported summary statistics |
| **United Kingdom** | Balp et al.^4^ | Government data^6^ | Age categories were derived from Gamma-simulated distributions based on reported summary statistics |
| **Italy** | Balp et al.^4^ | United Nations data^2^ | Age categories were derived from Gamma-simulated distributions based on reported summary statistics |
| **Japan** | Hide et al.^7^ | United Nations data^2^ | Age categories were derived from Gamma-simulated distributions based on reported summary statistics |
| **Korea** | Lee et al.^8^ | United Nations data^2^ | Proportion-based estimates were derived by normalizing category frequencies to cohort-specific sample sizes |
| **Spain** | Balp et al.^4^ | Government data^9^ | Age categories were derived from Gamma-simulated distributions based on reported summary statistics |
| **United States of America** | Soong et al. ^10^ | United Nations data^2^ | Age categories were derived from Gamma-simulated distributions based on reported summary statistics |

**Supplementary Table 1. Sources used for MRP estimates and used methodology for each country**

| **Variable** | **Brazil** | **China** | **France** | **Germany** | **Italy** | **Japan** | **Korea** | **Spain** | **UK** | **USA** |
| --- | --- | --- | --- | --- | --- | --- | --- | --- | --- | --- |
| ***N* participants (n)** | 1746 | 122 | 253 | 4473 | 438 | 277 | 468 | 799 | 1547 | 2120 |
| **Age (mean, SD)** | 37.5 (12.0) | 37.6 (12.3) | 38.2 (13.3) | 40.0 (13.1) | 42.1 (13.3) | 41.1 (12.4) | 38.7 (11.1) | 39.3 (13.0) | 37.7 (12.8) | 41.0 (13.7) |
| **Female (n, %)** | 1385 (79.3) | 85 (69.7) | 199 (78.7) | 3395 (75.9) | 309 (70.5) | 183 (66.1) | 310 (66.2) | 570 (71.3) | 1112 (71.9) | 1533 (72.3) |
| **Reporting of perceived aggravating factors (n, %)** | 1477 (84.6) | 66 (54.1) | 158 (62.5) | 2607 (58.3) | 269 (61.4) | 110 (39.7) | 202 (43.2) | 470 (58.8) | 954 (61.7) | 1286 (60.7) |
| **Exercise** | 191 (10.9) | 11 (9.0) | 36 (14.2) | 412 (9.2) | 44 (10.0) | 16 (5.8) | 43 (9.2) | 65 (8.1) | 265 (17.1) | 241 (11.4) |
| **Low temperature** | 108 (6.2) | 8 (6.6) | 28 (11.1) | 360 (8.0) | 28 (6.4) | 11 (4.0) | 46 (9.8) | 58 (7.3) | 208 (13.4) | 165 (7.8) |
| **High temperature** | 381 (21.8) | 14 (11.5) | 54 (21.3) | 786 (17.6) | 76 (17.4) | 30 (10.8) | 57 (12.2) | 141 (17.6) | 446 (28.8) | 494 (23.3) |
| **Rubbing of the skin** | 422 (24.2) | 22 (18.0) | 63 (24.9) | 1054 (23.6) | 100 (22.8) | 39 (14.1) | 69 (14.7) | 139 (17.4) | 370 (23.9) | 522 (26.0) |
| **Food-related** | 285 (16.3) | 23 (18.9) | 47 (18.6) | 738 (16.5) | 92 (21.0) | 23 (8.3) | 74 (15.8) | 79 (9.9) | 295 (19.1) | 374 (17.6) |
| **Medication** | 135 (7.7) | 11 (9.0) | 33 (13.0) | 484 (10.8) | 63 (14.4) | 7 (2.5) | 21 (4.5) | 52 (6.5) | 122 (7.9) | 167 (7.9) |
| **Infection** | 135 (7.7) | 6 (4.9) | 32 (12.6) | 521 (11.6) | 31 (7.1) | 3 (1.1) | 12 (2.8) | 32 (4.0) | 177 (11.4) | 149 (7.0) |
| **Stress** | 653 (37.4) | 27 (22.1) | 107 (42.3) | 1273 (28.5) | 133 (30.4) | 59 (21.3) | 110 (23.5) | 242 (30.3) | 524 (33.9) | 680 (32.1) |
| **Angioedema (n, %)** | 975 (55.8) | 44 (36.1) | 133 (52.6) | 2345 (52.4) | 214 (48.5) | 54 (19.5) | 139 (29.7) | 270 (33.8) | 870 (56.2) | 961 (45.3) |
| **Wheals appearing every day (n, %)** | 742 (44.8) | 23 (21.3) | 104 (44.1) | 1622 (38.5) | 220 (52.6) | 69 (27.0) | 92 (21.5) | 250 (32.9) | 648 (45.6) | 849 (42.7) |
| **Baseline UCT (median, IQR)** | 6 (7) | 8 (6) | 6 (6) | 6 (5) | 6 (6) | 8 (6) | 8 (5) | 7 (6) | 5 (6) | 5 (5) |

**Supplementary Table 2. Characteristics of the analyzed sample grouped by country**

SD - Standard deviation; UK - United Kingdom; UCT - Urticaria Control Test; USA - United States of America

**Supplementary Table 3. Prevalence of individual perceived aggravating factors among users reporting at least one aggravating factor**

| **Variable** | **Brazil** | **China** | **France** | **Germany** | **Italy** | **Japan** | **Korea** | **Spain** | **UK** | **USA** |
| --- | --- | --- | --- | --- | --- | --- | --- | --- | --- | --- |
| **Reporting of perceived aggravating factors (%, 95% CI)** |  |  |  |  |  |  |  |  |  |  |
| **Exercise** | 13.2 (10.4–16.6) | 14.8 (11.9–18.3) | 21.9 (17.4–27.0) | 19.5 (16.1–23.2) | 18.0 (14.9–21.7) | 13.9 (10.1–18.7) | 17.7 (10.6–25.9) | 16.6 (13.8–19.7) | 28.5 (24.3–33.0) | 22.6 (17.6–28.1) |
| **Low temperature** | 7.1 (5.1–9.9) | 11.3 (8.2–15.0) | 17.0 (12.2–22.7) | 14.2 (10.4–18.5) | 10.3 (7.1–14.5) | 9.8 (5.7–15.9) | 18.9 (12.4–27.9) | 12.2 (8.8–16.2) | 20.8 (15.5–26.1) | 13.3 (8.2–19.6) |
| **High temperature** | 25.5 (22.7–28.2) | 23.1 (20.6–25.9) | 33.4 (29.3–37.5) | 31.3 (27.7–35.2) | 28.2 (24.2–32.6) | 26.2 (20.7–32.4) | 26.9 (19.3–37.4) | 29.8 (26.2–33.4) | 45.6 (41.1–49.7) | 40.5 (27.1–53.9) |
| **Rubbing of the skin** | 27.0 (21.7–33.0) | 31.1 (25.2–37.6) | 35.9 (29.5–43.0) | 36.3 (31.3–41.6) | 32.4 (27.4–38.4) | 32.1 (24.8–40.0) | 32.0 (23.4–42.0) | 26.1 (22.0–30.3) | 36.3 (30.9–41.8) | 40.6 (36.1–45.2) |
| **Food-related** | 19.2 (14.5–24.4) | 30.5 (24.8–36.8) | 27.8 (21.8–34.6) | 26.9 (21.6–33.3) | 31.9 (24.3–40.4) | 22.1 (14.7–30.7) | 34.2 (25.6–43.6) | 17.0 (13.0–22.3) | 30.5 (23.6–37.7) | 28.7 (17.9–42.3) |
| **Infection** | 8.5 (4.2–15.7) | 8.7 (4.6–15.6) | 17.3 (9.9–28.0) | 16.5 (10.3–26.0) | 9.4 (4.6–17.8) | 4.5 (1.6–11.3) | 6.7 (3.0–13.4) | 5.7 (3.0–10.3) | 16.7 (9.8–27.3) | 10.3 (5.7–17.6) |
| **Medication** | 19.3 (13.3–27.2) | 16.2 (10.8–23.2) | 18.5 (12.1–26.2) | 15.8 (11.3–22.3) | 20.0 (13.6–28.1) | 9.9 (5.2–16.9) | 12.1 (6.8–19.3) | 9.9 (6.4–14.7) | 11.9 (7.8–18.1) | 11.3 (7.9–15.5) |
| **Stress** | 42.6 (38.6–46.7) | 42.1 (38.1–46.6) | 60.3 (55.8–64.7) | 46.8 (43.0–50.8) | 46.4 (42.3–50.9) | 49.1 (42.8–55.4) | 50.5 (41.0–60.3) | 48.1 (44.4–51.8) | 53.6 (49.6–57.6) | 52.1 (45.7–59.2) |

CI - Confidence Interval; UK - United Kingdom; USA - United States of America

**Supplementary References**

1.	Balp MM, Lopes da Silva N, Vietri J, Tian H, Ensina LF. The Burden of Chronic Urticaria from Brazilian Patients’ Perspective. *Dermatol Ther (Heidelb)*. 2017;7(4):535-545. doi:10.1007/s13555-017-0191-4

2.	United Nations | Population Division Data Portal. population.un.org/dataportal/home

3.	Zhang X, Song X, Zhang M, et al. Prevalence and risk factors of chronic urticaria in China: A nationwide cross-sectional study. *Allergy*. 2022;77(7):2233-2236. doi:10.1111/all.15287

4.	Balp MM, Krupsky K, Gupta S, et al. Prevalence, treatment and burden of chronic spontaneous urticaria in five European countries. *Journal of the European Academy of Dermatology and Venereology*. 2025;39(10):1806-1817. doi:10.1111/jdv.20772

5.	Pyramides des âges − Bilan démographique 2022 | Insee. www.insee.fr/fr/statistiques/6688661

6.	Estimates of the population for the UK, England, Wales, Scotland, and Northern Ireland - Office for National Statistics. ons.gov.uk/peoplepopulationandcommunity/populationandmigration

7.	Hide M, Uda A, Maki F, et al. Prevalence and Burden of Chronic Spontaneous Urticaria in Japan: A Cross-Sectional Study. *J Clin Med*. 2025;14(4). doi:10.3390/jcm14041162

8.	Lee N, Lee JD, Lee HY, Kang DR, Ye YM. Epidemiology of Chronic Urticaria in Korea Using the Korean Health Insurance Database, 2010-2014. *Allergy Asthma Immunol Res*. 2017;9(5):438-445. doi:10.4168/aair.2017.9.5.438

9.	Resident population by date, sex, age group and nationality (Spanish/foreign). https://www.ine.es/jaxiT3/Tabla.htm?t=59587&L=1

10.	Soong W, Patil D, Rodrigues J, et al. Clinical profile, prevalence, and burden of chronic spontaneous urticaria in the United States. *World Allergy Organ J*. 2025;18(8):101081. doi:10.1016/j.waojou.2025.101081
